# Patients with chronic peripheral vestibular hypofunction compared to healthy subjects exhibit differences in gaze and gait behaviour when walking on stairs and ramps

**DOI:** 10.1371/journal.pone.0189037

**Published:** 2017-12-18

**Authors:** Jaap Swanenburg, Edith Bäbler, Rolf Adelsberger, Dominik Straumann, Eling D. de Bruin

**Affiliations:** 1 Physiotherapy and Occupational Therapy Research Center, Directorate of Research and Education, University Hospital Zurich, University of Zurich, Zurich, Switzerland; 2 Interdisciplinary Spinal Research ISR, Department of Chiropractic Medicine, Balgrist University Hospital, Zurich, Switzerland; 3 Institute of Human Movement Sciences and Sport, Department of Health Sciences and Technology, ETH Zurich, Zurich, Switzerland; 4 Wearable Computing Lab Zurich, ETH Zurich, Zurich, Switzerland; 5 Department of Neurology, University Hospital Zurich, University of Zurich, Zurich, Switzerland; Tokai University, JAPAN

## Abstract

**Objective:**

The aim of this study was to compare gaze behaviour during stair and ramp walking between patients with chronic peripheral vestibular hypofunction and healthy human subjects.

**Methods:**

Twenty four (24) patients with chronic peripheral vestibular hypofunction (14 unilateral and 10 bilateral) and 24 healthy subjects performed stair and ramp up and down walks at self-selected speed. The walks were repeated five times. A mobile eye tracker was used to record gaze behaviour (defined as time directed to pre-defined areas) and an insole measurement device assessed gait (speed, step time, step length). During each walk gaze behaviour relative to *i*) detection of first transition area “First TA”, *ii*) detection of steps of the mid-staircase area and the handrail “Structure”, *iii*) detection of second transition area “Second TA”, and *iv*) looking elsewhere “Elsewhere” was assessed and expressed as a percentage of the walk duration. For all variables, a one-way ANOVA followed by contrast tests was conducted.

**Results:**

Patients looked significantly longer at the “Structure” (p<0.001) and “Elsewhere” (p<0.001) while walking upstairs compared to walking downstairs (p<0.013). Patients looked significantly longer at the “Structure” (p<0.001) and “Elsewhere” (p<0.001) while walking upstairs compared to walking downstairs (p<0.013). No differences between groups were observed for the transition areas with exception of stair ascending. Patients were also slower going downstairs (p = 0.002) and presented with an increased step time (p = 0.003). Patients were walking faster up the ramp (p = 0.014) with longer step length (p = 0.008) compared to walking down the ramp (p = 0.050) with shorter step length (p = 0.024).

**Conclusions:**

Patients with chronic peripheral vestibular hypofunction differed in time directed to pre-defined areas during stair and ramp walking and looked longer at stair and ramp areas of interest during walking compared to healthy subjects. Patients did not differ in time directed to pre-defined areas during the stair-floor transition area while going downstairs, an area where accidents may frequently occur.

## Introduction

Negotiating stairs or ramps is a challenging activity of daily living and stairway falls appear particularly evident for middle-aged (the years between 45 and 65 or thereabout) adults [1–3] next to being a leading cause of accidental death among older adults (persons aged ≥65 years) [4]. Stair falls account for more than 10% of fatal fall accidents [4, 5]. Until recently, stairway accidents were mainly attributed to a flaw in design or construction; e.g. poor railing, slippery tread [6], poor lighting conditions. These construction flaws may explain occurrence of some falls, however, the loss of sensory and motor function, disease, and disability must also be considered as possible causes for falls on stairs [6]. Locomotion on stairs places demand on the musculoskeletal and cardiovascular systems that are compounded by the need for input from the somatosensory, visual, and vestibular systems at various stages during the locomotion on stairs task [4]. Thus, besides construction flaws impairments in physical functioning, e.g. through the loss of vestibular, visual, or somatosensory information processing, may lead to accidents during stair negotiation [6, 7].

A dysfunction of the vestibular system directly influences the ability to maintain postural control and the risk of falling [8–10]. Cohen (1992) observed that 75% of patients with peripheral vestibulopathy aged between 35 and 82 years had difficulty climbing stairs [11]. An epidemiological accident study of falls on stairs revealed that accidents on stairs occur more frequently during stair descent [12]. A 12-month prospective study investigating the occurrence of falling in patients with bilateral chronic peripheral vestibular hypofunction (cPVH) revealed that nearly half of the falls (45%) occurred during stair negotiation. One-third of these falls occurred while descending stairs and at the lowest step while transitioning from the stair to level ground walking [13]. A study investigating elderly suffering from cPVH reported the most frequent tasks performed at the moment of a fall were walking (5%), climbing up or down stairs (11%), changes of posture (9%) and taking a bath (6%) [14].

Horak and colleagues (2009) demonstrated that individuals may use different strategies to substitute for their loss of vestibular function. Some patients were using their vision better whereas others were dependent on light touch on a stable surface to substitute for absent vestibular information, and a third group would mainly use the remaining vestibular function to master locomotion tasks [15]. There are differences with regard to which extent compensation mechanisms in patients can be deployed [15, 16]. Visual information can be used to compensate for lack of vestibular information and is also needed to detect step boundaries, handrail location, and potential hazards during stair negotiation [6]. Visual information, thus, plays an important role for effective postural control during stair negotiation [17, 18] in patients with peripheral vestibulopathy.

A difference in gaze behaviour during stair negotiation was observed between young and older adults, where gaze behaviour is defined as the time directed to pre-defined areas [18]. Older participants spent significantly more time looking toward the travel path during the middle section of the staircase [18]. Other studies, however, speculated that individuals may rely on a spatial representation established from previous experience and/or visual information other than gaze fixations to extract information from the surrounding environment for safe stair negotiation [19]. The ability to sense upcoming transitions has been identified as an important factor in the evaluation of fall-prone or disabled individuals [20] and patients with bilateral cPVH report stair-to-ground transitions as possible cause for their stair negotiation difficulties [13].

To date, no studies have quantitatively assessed gaze and walking behaviour during locomotion on stairs or ramps in patients with bilateral cPVH. Information on gaze and walking behaviour specifically during stair and ramp negotiation is important to contribute to the thorough understanding of the underlying visuomotor control of stair and ramp walking and the effects of vestibular loss on postural control, specifically in patients with bilateral cPVH. Due to a lack of scientific literature on gaze and walking behaviour in patients with chronic peripheral vestibular hypofunction, there is limited understanding of how stair negotiation contributes to falling in people with cPVH. A complete understanding of underlying visuomotor control of stair and ramp walking may assist in the development of potential interventions and help to define important components these interventions should address; e.g. postural control, attention while negotiating stairs and ramps, for patients with cPVH.

The aims of the present study were to quantitatively describe where and when individuals look during stair and ramp negotiation and to determine whether there are any differences in these measures between patients with unilateral or bilateral cPVH and healthy subjects. This knowledge could contribute to our understanding of the increased incidence of stair and ramp falls in populations with vestibulopathy. We hypothesized that (a) both patients with unilateral or bilateral cPVH and healthy subjects would spend the majority of time looking at future stepping areas on the stairs and ramps, but that (b) patients would look to these locations differently compared to healthy subjects.

## Methods

### Participants

The study sample included a convenience sample of patients with cPVH and healthy control subjects. The inclusion criterion was patients with cPVH on one or both sides for two years or longer. One- or two-sided cPVH was diagnosed by a pathological horizontal head impulse test to both sides (< 0.68), as assessed by a video-based system [21] and the presence of saccades. Outpatients and inpatients with vestibulopathy were recruited in the Departments of Neurology and Otorhinolaryngology at University Hospital Zurich (USZ). A senior neurologist confirmed the inclusion criteria. Healthy subjects were recruited from the hospital staff and the greater area of Zurich community dwellers by personal invitation or email. All measurements were obtained at the hospital. Patients with Menière's disease or with benign paroxysmal positional vertigo were excluded. Additionally, excluded were patients with (1) need of walking aid, (2) drugs or substance abuse (in particular sedatives, anti-depressants, anti-epileptics, neuroleptica and/or alcohol) possibly interfering with gait, (3) gait disorders putatively attributed to other than primarily vestibular causes, cerebral pathologies such as neurodegenerative disorders, stroke, spinal pathologies; e.g. mass lesion, vitamin deficiency, and/or peripheral neuropathy. All participants provided written informed consent prior to commencement of assessment.

A total of 24 consecutively recruited patients with cPVH (mean age of 63.2, SD 12.8years, 8 male) were analysed. Fourteen patients were categorised having a unilateral and 10 having a bilateral vestibular disorder. The causes of vestibular disorder included seven with vestibular neuritis, three patients taking ototoxic medications, two with schwannoma, and 12 patients with unknown aetiologies. Additionally, 24 healthy subjects (mean age: 56.0, SD 13.8years, 10 male) were recruited.

All but one patient had experienced any kind of vertigo during measurement. The study was approved by the ethics committee of the Canton of Zürich under KEK-ZH-NR: 2014–0509. ClinicalTrials.gov Identifier; NCT02417545

### Procedure

Assessment started with stair walks followed by ramp walks. Before each walk a cardboard (35cm x 50cm) was held in front of each participant’s face to prevent any early visual exploration of the staircase or ramp environment [19]. All participants were asked to start walking when the cardboard was moved aside. Participants were asked to walk at their preferred speed and to start moving with their preferred leg while using the handrail was discouraged. Participants that felt they had to use the handrail were told to not use the handrail to pull themselves upward on the stairs or ramp but rather use it as a guidance by lightly touching it. For security reasons a therapist walked behind the patients. Participants were asked to perform 5 repetitions of each task to account for possible learning effects and allow familiarisation with the tasks. In between stair walks, a 30 second break was implemented. Different recommendations from previous research for analysing gaze behaviour during stair negotiation; the use of a staircase with at least five steps [22], a particular focus on stair descent [6], and inclusion of inclined surfaces or ramps [5, 23, 24], were explicitly considered in our measurement procedure.

Stairs

**Fig 1:**
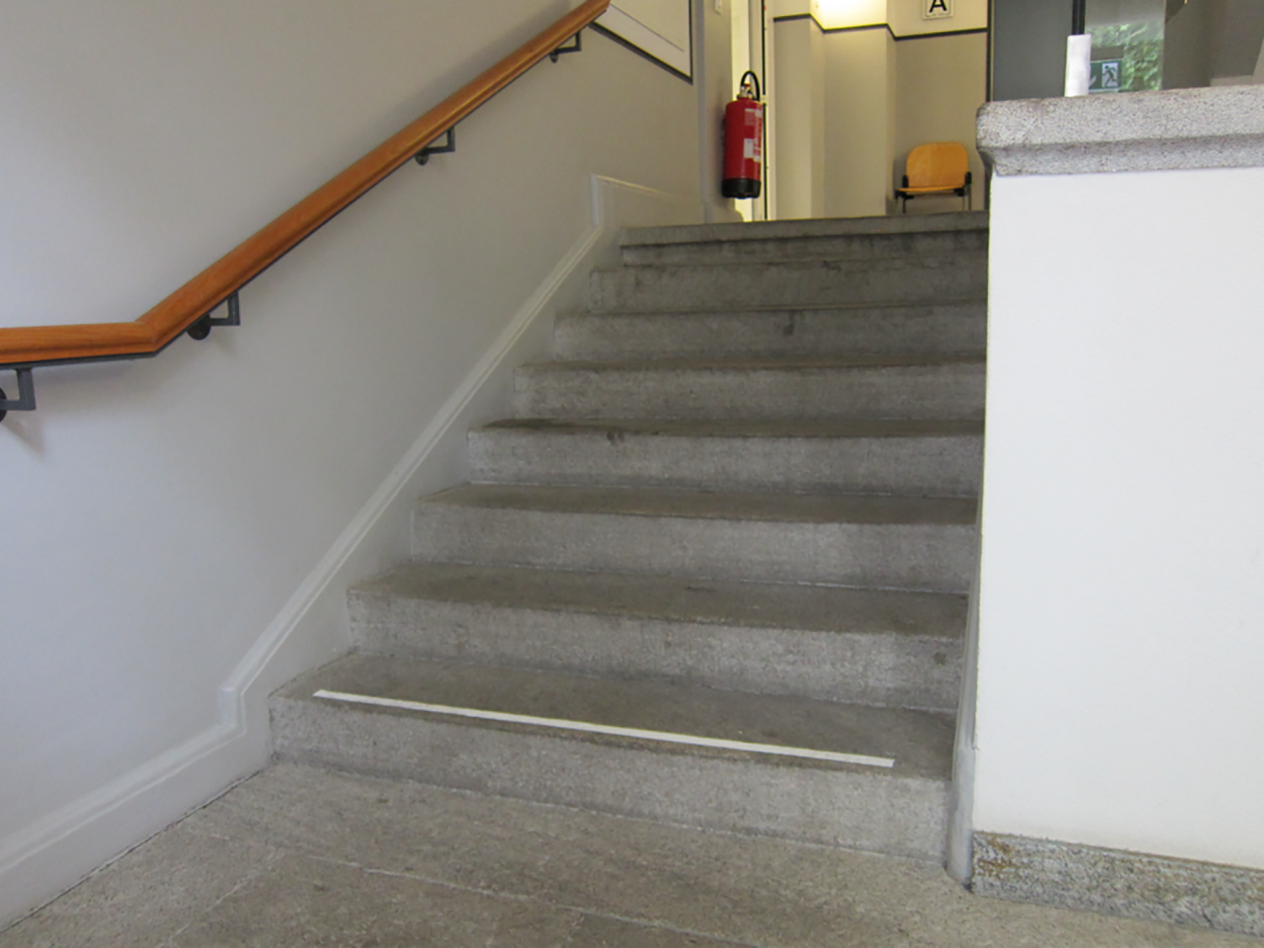
Stairs looking upwards.

**Fig 2:**
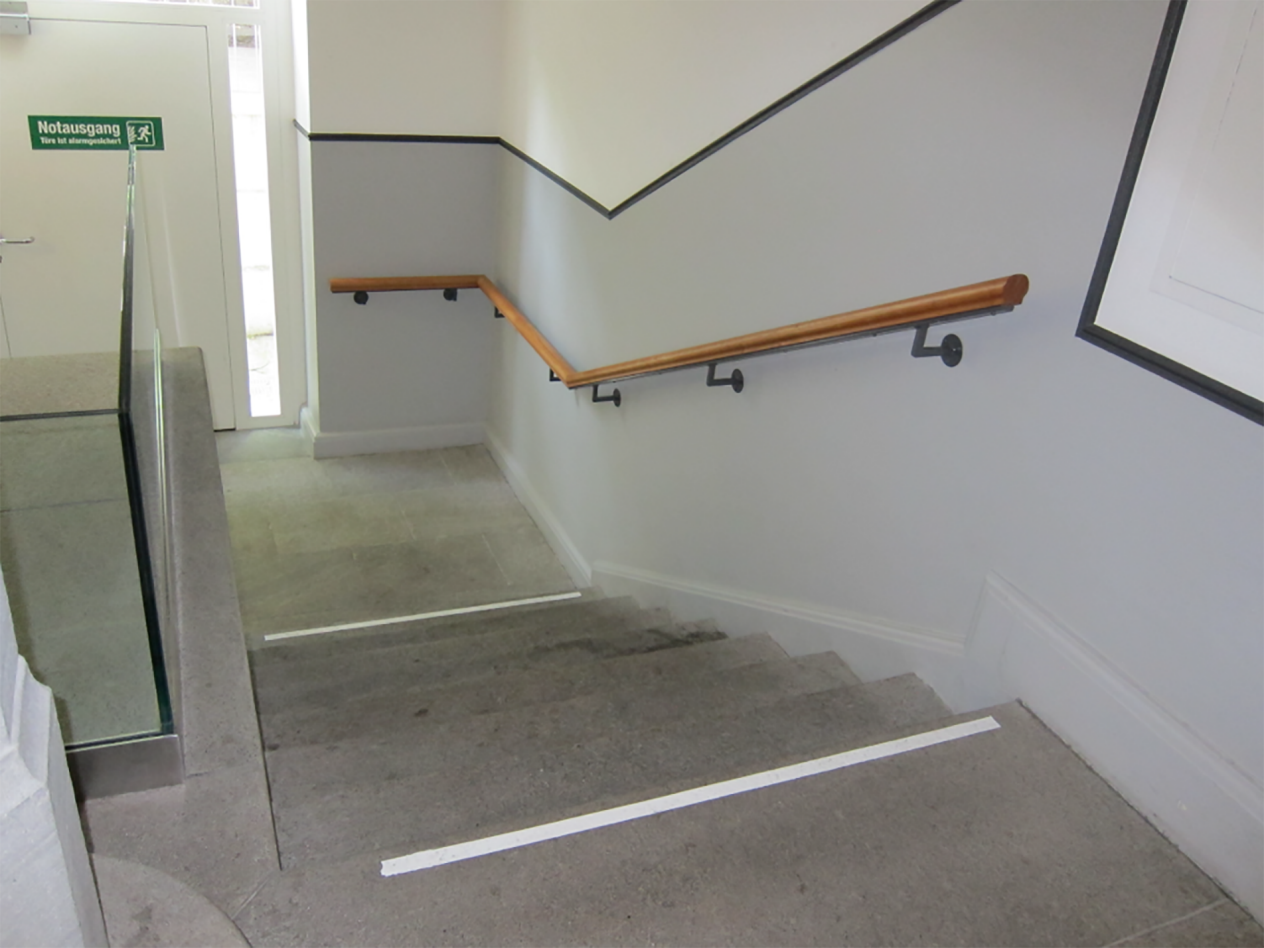
Stairs looking downwards.

The stair-walking task always started with stair descent. The starting position was one meter in front of the staircase (step height 15cm, step length 30 cm, according legislation standards and recommendations DIN 18065) with eight steps, thus, allowing approaching gait before stair negotiation [25]. End position was one meter after the last step and a handrail was located on the right side when descending (Figs 1 and 2).

Ramp

**Fig 3:**
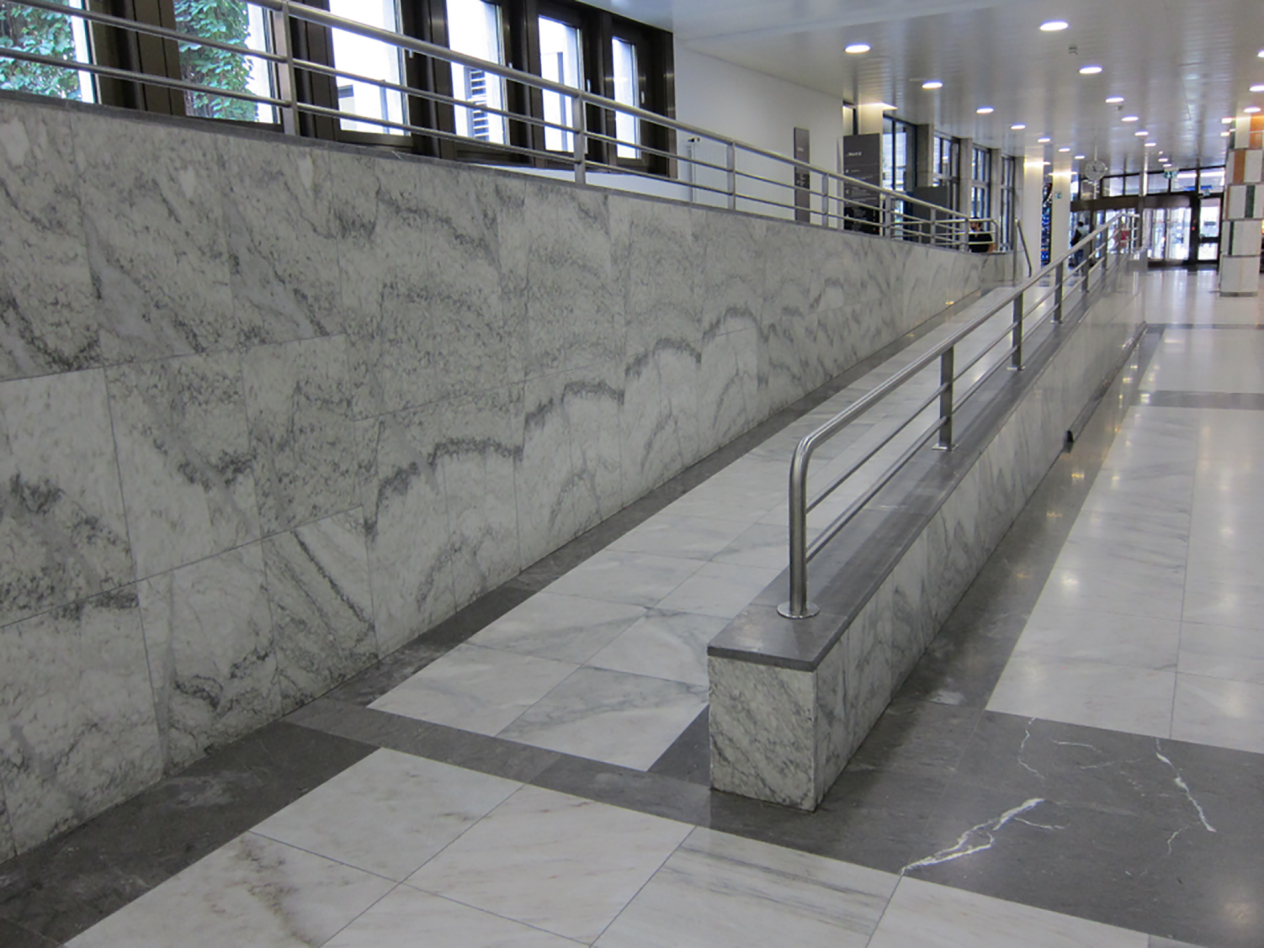
Ramp looking upwards.

**Fig 4:**
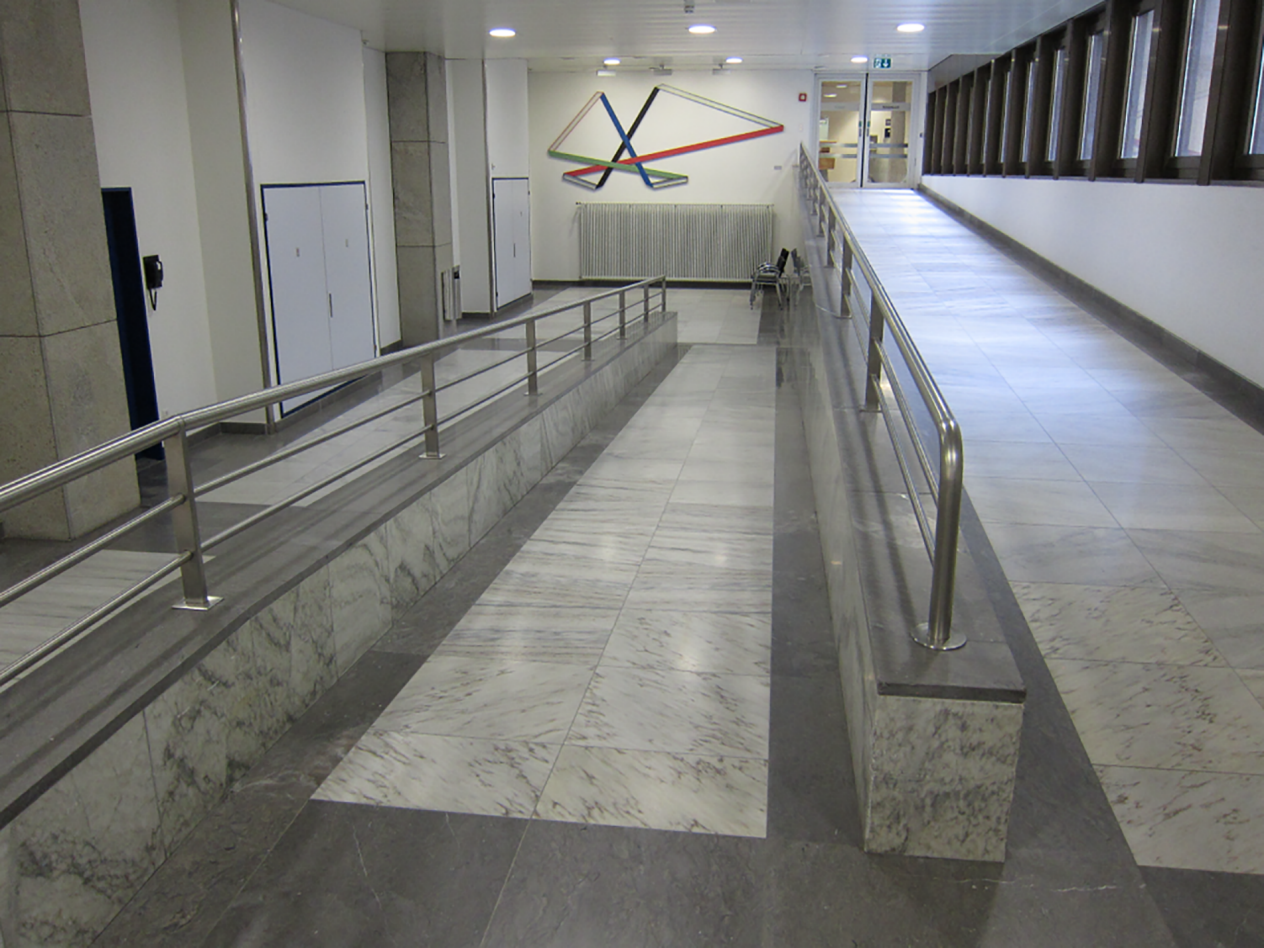
Ramp looking downwards.

For the ramp walking all participants were asked to start with ramp ascent. The starting position was one meter in front of the ramp. End position was one meter after the ramp. Pitch ratio in percent of the ramp was 5.9% (0.7m:11.8m) and a handrail was located on the right side when descending. The maximum pitch should be no more than 6% according legislation standards and recommendations (Figs 3 and 4).

### Gaze assessments

A mobile eye tracker (Dikablis Professional Glasses, Ergoneers Inc., Manching, Germany) was used to record gaze behaviour during the stairs and ramp negotiation. The mobile setup consisted of a head and a processing unit. The head unit contains three cameras: two directed to the subject's eyes to record the eye movements, and one directed to the gait scene. Eye camera tracking frequency(s) was 60 Hz (per eye). The pupil tracking accuracy is 0.05° visual angle, and glance direction accuracy 0.1° - 0.3° visual angle. Participants wore the equipment over their glasses when needed. The Dikablis Mobile system is only minimally interfering with a participant’s natural viewing behaviour [26] and can discriminate gaze behaviour between young and older subjects [27, 28].

Next to the head unit, the eye tracker was connected to a processing unit that consisted of a receiver and a recording tablet computer. The processing unit was stored in a backpack worn by the participant while walking on the stairs and ramp. The mobile setup was connected to the recording notebook via Wi-Fi. The D-Lab 3.01 software package (Ergoneers Inc, Manching, Germany) on a notebook was used for data recording, monitoring, and processing. For this study the mean of both recordings (left and right eye) were used.

Gaze data were analysed frame by frame with D-Lab 3.01 software. Areas of interest (AoI) were defined both for the stairs and ramp. For each walk gaze behaviour (dependent variable time directed to each AoI; e.g. the cumulative glances duration distribution across the AOIs, divided by the total glances duration time calculated for each participant and each walk) was expressed as a percentage of the walk duration. Because the investigators were especially interested in visual behaviour at transitional zones, we defined different AoI;

Stair AoI

**Fig 5:**
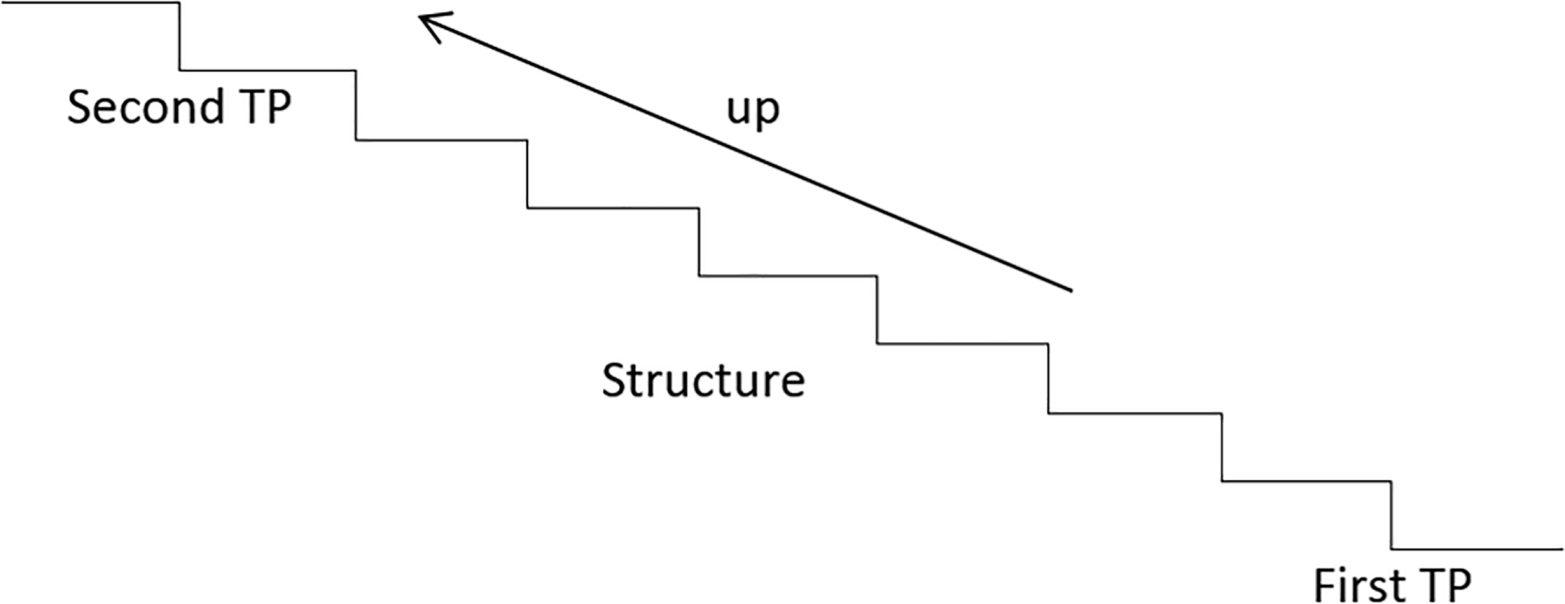
Areas of interest stair looking upwards.

**Fig 6:**
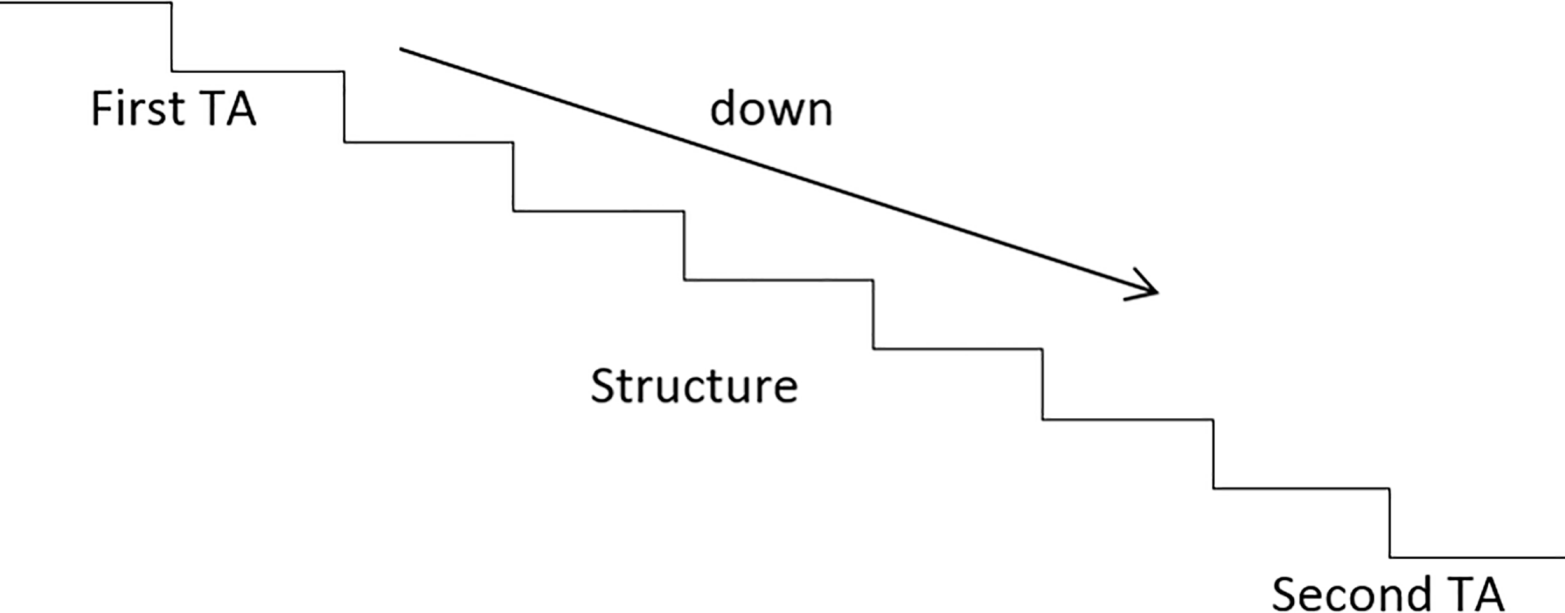
Areas of interest stair looking downwards.

- First transition area (First TA) included one tread-length before the first step and the first step
- Structure area (Structure) includes the five steps of the mid-stair region and the handrail.
- Second transition area (Second TA) included the last step and one tread-length after the stair.
- Looking elsewhere (Elsewhere); the area where vision was not directed to the stair structure and/or transition areas (Figs 5 and 6).

Ramp AoI

**Fig 7:**
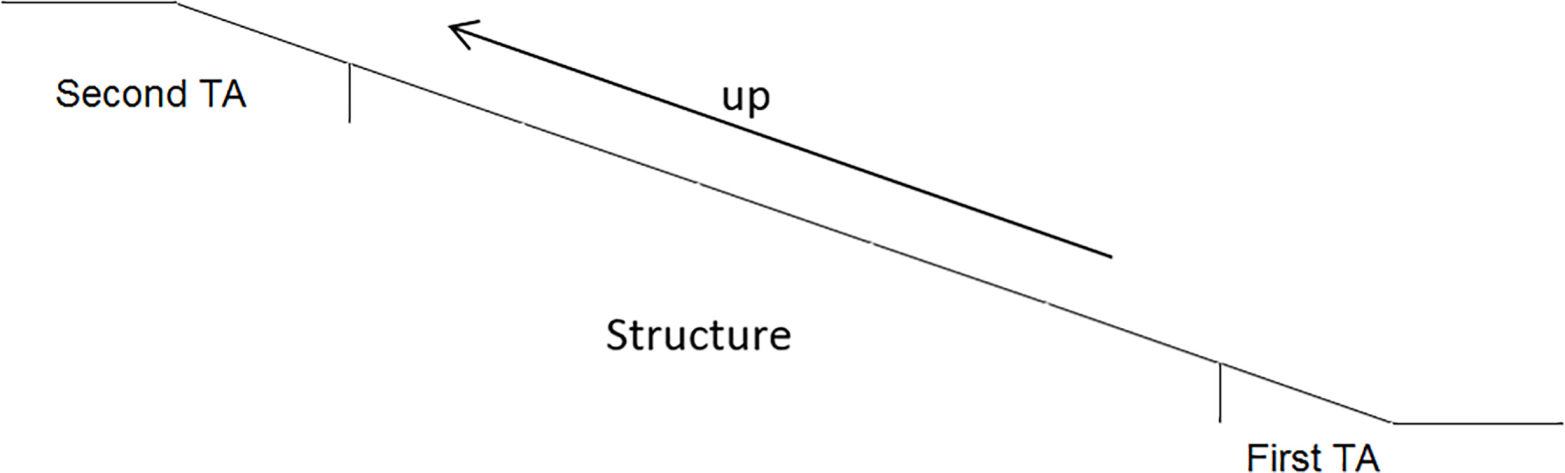
Areas of interest ramp looking upwards.

**Fig 8:**
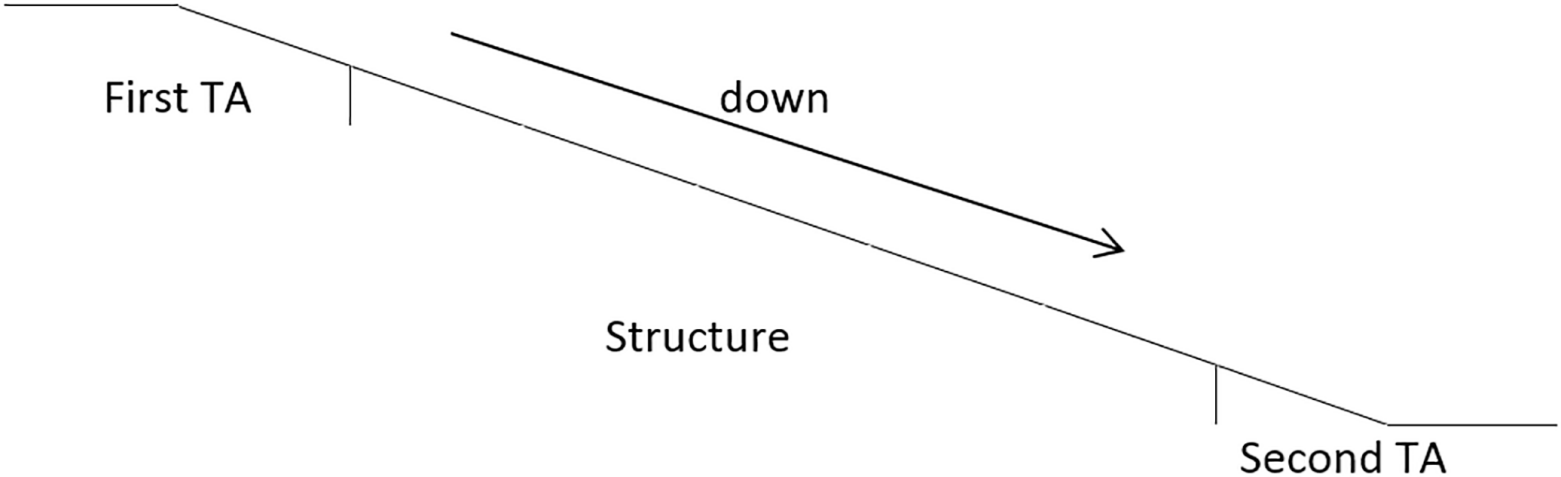
Areas of interest ramp looking downwards.

- First transition area (First TA) included 1m before and after the transition point.
- Structure area (Structure) includes the middle part of the ramp and the handrail.
- Second transition area (Second TA) 1m before and after the transition point.
- Looking elsewhere (Elsewhere), the area where vision is not directed to the ramp structure and/or transition areas (Figs 7 and 8).

### Gait assessments

**Fig 9:**
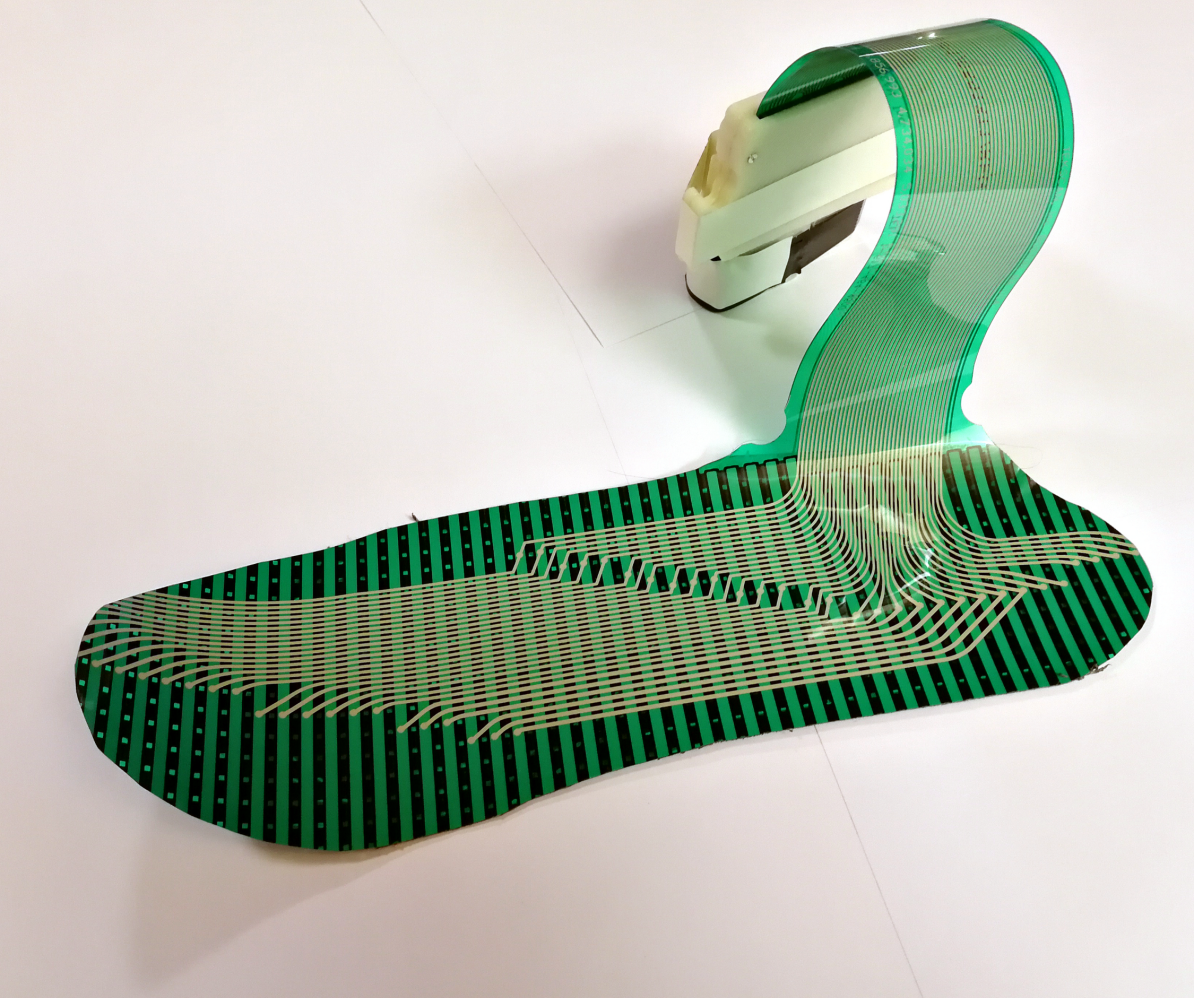
Insole gait measurement device.

Gait variables during stair/ramp walking were assessed with an insole measurement device. This device has an inertial measurement unit (IMU) and a force-sensitive plastic foil (Fig 9). The IMU recorded acceleration and rotation rates in each three dimensions. The foil has more than 1200 force-sensitive resistors [29], and was placed in both shoes of the participant. The IMU was taped above the ankle. The participants were asked to make a few steps to check if the device was not hindering them. All data were stored on the SD cards of the sensors for off-line analysis. The system can discriminate between healthy subjects and subjects with reduced postural stability with an accuracy of 94% [30]. The following gait variables were derived from the measurements: Gait speed and mean step time were calculated from foot-contact to foot-contact for each trial and for the different AoI separately. Additionally, for ramp negotiation mean step length and step length were calculated.

### Statistics

Descriptive statistics were used to describe the study population and to calculate stair and ramp negotiation characteristics. The mean of five walk trials was used for further calculations. To identify differences between groups in all variables planned contrasts analysis were used [31]. A one-way ANOVA followed by planned contrasts between healthy controls and all patients in a first step was used for data analysis. This was followed by a second step in which unilateral vs bilateral vestibular disorder patients were compared. The significance level of the statistical analysis was set at p<0.05. If the tests of homogeneity of variances (Levene Statisics) were significant, results for unequal variance of the contrast test were used. The data were entered, stored, and analysed using IBM-SPSS 22 statistical software (SPSS, Chicago, IL).

## Results

Twelve patients and two healthy subjects had to use the handrail during stair negotiation. During ramp negotiation, one patient and one healthy individual used the handrail. Six patients walked in the middle of the stairs and 18 on the side close to the handrail. Twenty healthy subjects were walking in the middle of the stairs. There was no significant difference between groups for age even though the controls were younger on average. The participants’ characteristics are summarized in Table 1.

**Table 1:** Descriptive statistics summarizing the patients’ and healthy subjects’ characteristics.

	Healthy subjects; n = 24	Patients; n = 24			
		All	UV	BV	p
Female	14	16	10	6	na
Male	10	8	4	4	na
Age; years (SD)	56.0 (13.8)	63.2 (12.8)	60.0 (12.9)	67.9 (12.8)	0.068
Range	33/77	35/79	39/79	35/77	na
Weight; kg (SD)	71.1 (12.3)	71.5 (16.3)	73.7 (18.4)	68.4 (12.8)	0.929
Height; cm (SD)	170 (9.6)	165.7 (9.0)	168.2 (9.4)	162.2 (7.5)	0.109
Vertigo; count (%)	0 (0%)	23 (96%)	13 (93%)	10 (100%)	
Use of stair handrail; count (%)	2 (8%)	12 (50%)	6 (43%)	6 (60%)	
Use of ramp handrail; count (%)	1 (4%)	2 (8%)	1 (7%)	0 (0%)	
Walking stairs; Middle; count (%) Side; count (%)	20 (83%) 4 (17%)	6 (25%) 18 (75%)	4 (29%) 10 (71%)	2 (20%) 8 (80%)	
Walking ramp; Middle; count (%) Side; count (%)	20 (83%) 4 (17%)	18 (75%) 6 (25%)	11 (79%) 3 (21%)	7 (70%) 3 (30%)	

SD = standard deviation; kg = kilograms; cm = centimetres; p = significant difference (p<0.05) Mann-Whitney test; na = not applicable; UV = unilateral vestibular loss; BV = bilateral vestibular loss

### Gaze behaviour stair

The mean values and standard deviations of 48 evaluated patients and healthy subjects are reported in Table 2 for their grouping. Planned contrasts showed that there were significant differences in time directed to pre-defined areas on all AoI going up the stairs. There were significant effects of grouping between patients and healthy subjects on “Structure” t(45) = 3.942, p<0.001, “Elsewhere” t(45) = -3.913, p<0.001, and the second TA”, t(45) = 2.832, p = 0.007. Between unilateral and bilateral vestibular cPVH patients there was a significant effect during the “First TA” t(45) = 2.532, p = 0.015 and “Structure” t(45) = -2.395, p = 0.021. Going down stairs planned contrasts showed that there were significant differences in gaze behaviour at the AOI’s “Structure” and “Elsewhere”. There were significant differences between patients and healthy subjects on “Structure” t(45) = 2.601, p = 0.013 and “Elsewhere” t(45) = -2.591, p = 0.013. All results are shown in Table 2.

**Table 2:** Difference in gaze behaviour at the areas of interest going Up and DOWN stairs /ramp between healthy subjects and patients (unilateral and bilateral cPVH).

	Healthy subjects	Patients			one-way ANOVA		Contrast 1		Contrast 2	
	n = 24	All n = 24	UV n = 14	BV n = 10						
AoI	Mean (SD)	Mean (SD)	Mean (SD)	Mean (SD)	F(2,45)	p	t (45)	p	t (45)	p
**Stairs UP**										
First TA; % s	5.5 (4.8)	5.9 (4.8)	7.9 (4.9)	3.1 (3.2)	3.256	0.048*	0.015	0.988	2.532	0.015*
Structure; % s	27.6 (7.9)	35.6 (8.0)	32.5 (8.1)	40.0 (5.6)	9.654	<0.001*	3.942	<0.001*	-2.395	0.021*
Second TA; % s	13.1 (4.4)	16.5 (4.5)	15.6 (4.2)	17.8 (4.7)	4.370	0.018*	2.832	0.007*	-1.179	0.245
Elsewhere; % s	53.5 (13.1)	41.4 (9.3)	43.3 (10.8)	38.7 (6.2)	7.293	0.002*	-3.913	<0.001^†^*	1.313	0.203^†^
**Stair DOWN**										
Fist TA; % s	6.6 (6.1)	7.8 (5.8)	9.1 (5.7)	6.0 (5.7)	1.062	0.354	0.547	0.587	1.276	0.208
Structure; % s	31.4 (13.1)	39.7 (8.1)	39.5 (8.3)	39.9 (8.4)	3.407	0.042*	2.601	0.013*	-0.116	0.930
Second TA; % s	10.5 (5.2)	12.1 (5.2)	11.4 (4.7)	13.1 (5.9)	0.889	0.418	1.168	0.249	-0.777	0.441
Elsewhere; % s	51.4 (21.0)	39.1 (10.9)	40.2 (10.6)	37.6 (11.8)	3.320	0.045*	-2.591	0.013^†^*	0.560	0.582^†^
**Ramp UP**										
Fist TA; % s	1.5 (1.6)	2.0 (2.2)	2.1 (2.3)	1.70 (20.6)	0.556	0.641	0.265	0.792	0.935	0.355
Structure; % s	22.5 (15.4)	43.7 (22.1)	40.8 (22.1)	47.8 (22.4)	7.793	0.001 *	3.925	<0.001*	0.889	0.379
Second TA; % s	11.5 (8.6)	11.9 (8.1)	10.6 (7.0)	13.8 (9.4)	0.449	0.578	0.825	0.414	-0.554	0.582
Elsewhere; % s	64.3 (20.5)	42.2 (23.4)	16.2 (23.6)	36.6 (23.2)	6.636	0.003*	-3.587	0.001*	-1.056	0.296
**Ramp DOWN**										
Fist TA; % s	0.8 (1.4)	1.2 (1.8)	1.4 (1.6)	0.9 (2.0)	0.624	0.541	0.698	0.489	-0.783	0.438
Structure; % s	24.2 (15.6)	46.5 (20.2)	43.6 (20.0)	50.5 (20.9)	9.537	<0.001*	4.349	<0.001*	0.916	0.365
Second TA; % s	11.5 (8.6)	11.9 (8.1)	10.6 (7.0)	13.8 (9.4)	0.449	0.641	0.265	0.797	0.919	0.372
Elsewhere; % s	59.5 (21.6)	37.2 (20.2)	40.9 (20.4)	32.0 (19.8)	7.335	0.002*	-3.786	<0.001*	-1.023	0.312

% s = seconds; AoI = arias of interest

^†^ Sig. Test of Homogeneity of Variances = unequal variances

* = significant p<0.05

Contrast 1 = healthy subjects and vestibulopathy patients; Contrast 2 = patients with unilateral cPVH (UV) and bilateral cPVH (BV)

### Gaze behaviour ramp

Planned contrasts showed that there were significant differences in time directed to pre-defined areas AOI’s “Structure” and “Elsewhere”. There were significant differences between patients and healthy subjects on “Structure” t(45) = 3.925, p<0.001, and “Elsewhere” t(45) = -3.587, p = 0.001.

Going down the ramp, planned contrasts showed that there were significant differences in time directed to pre-defined areas on the AOI’s “Structure” and “Elsewhere”. There were significant effects of grouping between patients and healthy subjects on “Structure” t(45) = 439, p<0.001 and “Elsewhere” t(45) = -3.786, p<0.001. All results are shown in Table 2.

### Gait variables

The data of 22 patients and 22 healthy subjects were used for analysis. The data of four participants were not available while not recorded due to technical issues. Patients were going slower downstairs (p = 0.002) with longer step time (p = 0.003).

At the ramp, patients were faster walking up and down the ramp (p = 0.014/p = 0.050) and had longer step length (up p = 0.008; down p = 0.024). All gait results are shown in Table 3.

**Table 3:** Difference in gait variables going DOWN and UP stairs /ramp between healthy subjects and patients (unilateral and bilateral cPVH).

	Healthy subjects	Patients			one-way ANOVA		Contrast 1		Contrast 2	
	n = 22	All n = 22	UV n = 12	BV n = 10	F(2,41)	p	t (41)	p	t (41)	p
	Mean (SD)	Mean (SD)	Mean (SD)	Mean (SD)						
**Stairs UP**										
Mean gait speed; m/s	0.71 (0.08)	0.63 (0.10)	0.63 (0.08)	0.63 (0.12)	3.0165	0.053	0.950	0.347	0.017	0.987
Mean step time; s	1.01 (0.13)	1.14 (0.17)	1.13 (0.15)	1.15 (0.21)	2.913	0.066	-1.102	0.277	0.194	0.847
Mean step time Second TA; s	1.10 (0.72)	1.22 (0.19)	1.22 (0.16)	1.23 (0.24)	2.080	0.138	-0.981	0.332	0.219	0.827
**Stairs DOWN**										
Mean gait speed; m/s	0.92 (0.17)	0.73 (0.14)	0.73 (0.14)	0.72 (0.15)	7.160	0.002*	1.588	0.120	-0.158	0.883
Mean step time; s	0.80 (0.16)	1.01 (0.20)	1.00 (0.19)	1.02 (0.21)	6.650	0.003*	-1.609	0.115	0.205	0.819
Mean step time Second TA; s	0.85 (0.16)	1.03 (0.20)	1.03 (0.19)	1.02 (0.21)	5.588	0.007*	-1.223	0.228	-0.065	0.949
**Ramp UP**										
Mean gait speed; m/s	10.9 (0.16)	0.94 (0.17)	0.96 (0.15)	0.92 (0.23)	4.806	0.014*	1.552	0.129	-0.431	0.669
Mean step time; s	1.04 (0.06)	1.08 (0.08)	1.10 (0.07)	1.04 (0.09)	4.074	0.024*	1.435	0.159	-2.273	0.202
Mean step time Second TA; s	1.05 (0.13)	1.12 (0.10)	1.15 (0.09)	1.07 (0.08)	3.414	0.043*	1.708	0.095	-1.603	0.117
Step length; m	0.56 (0.06)	0.51 (0.08)	0.52 (0.06)	0.47 (0.11)	5.554	0.008*	2.636	0.012*	-1.677	0.102
Step length Second TA; m	0.58 (0.08)	0.53 (0.10)	0.54 (0.09)	0.50 (0.13)	2.665	0.083	0.688	0.495	-1.603	0.117
**Ramp DOWN**										
Mean gait speed; m/s	1.08 (0.14)	0.96 (0.19)	0.97 (0.17)	0.94 (0.25)	3.225	0.050*	1.260	0.215	-0.351	0.728
Mean step time; s	1.02 (0.05)	1.04 (0.07)	1.05 (0.05)	1.02 (0.08)	1.439	0.249	0.712	0.481	-1.248	0.219
Mean step time Second TA; s	1.01 (0.12)	1.03 (0.13)	1.06 (0.05)	0.98 (0.21)	1.166	0.322	1.179	0.245	-1.461	0.152
Step length; m	0.55 (0.06)	0.49 (0.09)	0.52 (0.08)	0.47 (0.11)	4.091	0.024*	2.022	0.050*	-1.126	0.267
Step length Second TA; m	0.56 (0.72)	0.50 (0.11)	0.51 (0.11)	0.48 (0.12)	2.518	0.094	1.463	0.152	-0.586	0.870

s = seconds; m/s = meter/seconds; m = meter

* Sig. Test of Homogeneity of Variances = unequal variances

n = count; s = seconds; m = metres; Contrast 1 = healthy subjects and patients; Contrast 2 = patients with unilateral cPVH (UV) and bilateral cPVH (BV)

## Discussion

The aims of the present study were to quantitatively describe where and when individuals look during stair and ramp negotiation and to determine whether there are differences in these measures between patients with unilateral or bilateral cPVH and healthy subjects. This novel investigation might contribute to our understanding of the increased incidence of stair and ramp falls in populations with vestibulopathy. We hypothesized that (a) both patients and healthy subjects would spend the majority of time looking at future stepping areas on the stairs and ramps, but that (b) patients would look to these areas differently than would healthy subjects. During stair and ramp ascent and descent there was a significant difference in gaze behaviour observable between healthy subjects and patients with cPVH. Patients were significantly more focused on the structure (mid-stair/ramp area, and handrail) whereas healthy subjects spent more time looking elsewhere. Only going upstairs patients had more gaze time focused towards the second (upper) transition area. Patients with bilateral cPVH looked significantly shorter at the first transition area, but significantly longer on the stair structure compared to the patients with unilateral cPVH. Whether these results can be explained because the patients need additional sensory information to compensate for loss of vestibular information [32] should be investigated in future projects.

It can be hypothesised that, by looking longer at the stair or ramp structures, patients can overcome their loss of vestibular sensory information. Patients with bilateral cPVH have to compensate more than those with unilateral cPVH. Surprisingly, however, there was no difference in gaze behaviour found during the transition areas between these two patient groups and the healthy controls. Our results seem to confirm findings from a recent review stating little association between fixation durations and the use of a stair tread, for transitional and continuous steps, and little need for fixation at handrails [19, 25, 33, 34] as seen in healthy young adults [25]. For older adults, stair negotiation requires attention in order to exhibit better performance, which implies the need of facilitating the attentional focus when stairs are used, as also evidenced by cognitive dual-task costs for motor behaviour [25]. It can be speculated that one of the reasons for no difference between groups in gaze behaviour on transitions is attributable to a lack of compensatory increases in attentional focus required for these transitions in vestibulopathy patients. Attentional and oculomotor processes are tightly integrated at the neural level [35] and can be trained [36]. The foregoing would, for example, imply that interventions need to focus on multiple components and contain some form of cognitive-motor training. It must be emphasised, however, that currently the scientific literature is not sufficiently progressed in the sense that we completely understand how stair negotiation contributes to falling in people with vestibulopathy, nor is the literature developed enough to substantially inform potential interventions.

The lower transition area going downstairs has been reported to be a location for accidents [13]. Young and colleagues (2010) hypothesised that the central nervous system prioritizes the use of proprioceptive and visual systems to compensate for the loss of vestibular information [37]. It seems that exactly during the most difficult part of the stair or ramp negotiation there is no visual compensation for the reduced vestibular information. It can be speculated that this behaviour of non-adapted gaze behaviour may be the reason for the higher fall risk at stair-to-floor transition when going downstairs.

The rather short gaze time found during transition area for the healthy subjects as well for the patients are comparable with the results of an earlier study performed with healthy adults [33]. Miyasike-daSilva and McIlroy (2012) investigated the role of foveal vision during stair negotiation by using a dual-task paradigm to influence the ability to rely on foveal vision and reported an increase in gaze time during the mid-stair phase in their subjects.

In this study a faster gait speed was found during stair decent and ramp negotiation in patients with cPVH. A higher gait speed was also found during a clinical functional test performed on a computerized gait assessment system in patients with bilateral cPVH [38, 39]. An 8% increase in gait speed after rehabilitation is documented in patients with vestibulopathy [40, 41]. Other authors concluded with increased walking speed strategy patients are better able to suppress misleading vestibular signals [42–45].

In this setting, the stair negotiation seems to require more attention in patients with vestibulopathy than ramp negotiation. During stair negotiation, more patients walked closer to the stair handrail and touched it more times than walking on the ramp. The behaviour of patients during the ramp negotiation was similar to the healthy subjects. The slope of the ramp perhaps was likely not challenging enough for these patients [46], however, the ramp used in our study was built in accordance with legislation standards and recommendations. Another future area of research is to investigate gaze behaviour while walking on more steeper surfaces because this might show greater differences.

Another future area of research is whether or not there is clinical meaningfulness to these measures, or determining what value/cut-off might prompt a treatment decision on whether or not to intervene.

### Limitations

Some limitations of the study should be mentioned. The first relates to the measurement system that was used. The eye-tracker system showed to possess variable accuracy and reliability in people with Parkinson's disease and older adults [47]. Furthermore, although most eye movement analytical approaches are based on time-integrated measures; e.g. the average fixation duration, the number of fixations directed towards a specific region of interest, extracting these data is highly challenging. Dynamic walking activities require automated methods to compare eye-tracking data of different participant groups in order to be able identifying differences in common patterns of eye movements [26]. There is a clear need for new algorithms that are able to tackle these challenges related to the psychometric properties of the measurement system in the future.

Consequences of vestibular dysfunctions are generally triggered by head movements, transfers, and walking [48]. In this study, head movements were not assessed. Falls during the transition phase walking downstairs could also be triggered by an offset of consequences of vestibular dysfunctions. Future studies should also assess head movements in fallers with vestibulopathy during stair negotiation.

## Conclusion

Patients with chronic peripheral vestibular hypofunction differed in time directed to pre-defined areas during stair and ramp walking and looked longer at stair & ramp areas of interest during walking compared to healthy subjects. Patients did not differ in time directed to pre-defined areas during the lower stair-floor transition area while going downstairs, an area where accidents typically happen. Patients were walking slower going downstairs. At the ramp, patients were faster walking up and down.

## Supporting information

S1 Dataset Complete gaze data dataset on which the present study is based. (XLSX)

S2 Dataset Complete gait data dataset on which the present study is based. (XLSX)

## References

[1] Kool B, Ameratunga S, Hazell W, Ng A. Unintentional falls at home among young and middle-aged New Zealanders resulting in hospital admission or death: context and characteristics. N Z Med J. 2010;123(1316):75–84. . 20648101

[2] Malta DC, Silva MM, Mascarenhas MD, Sa NN, Morais Neto OL, Bernal RT, et al The characteristics and factors of emergency service visits for falls. Rev Saude Publica. 2012;46(1):128–37. . 22249755 10.1590/s0034-89102012000100016

[3] Talbot LA, Musiol RJ, Witham EK, Metter EJ. Falls in young, middle-aged and older community dwelling adults: perceived cause, environmental factors and injury. BMC Public Health. 2005;5:86 doi: 10.1186/1471-2458-5-86 ; PubMed Central PMCID: PMCPMC1208908. 16109159 10.1186/1471-2458-5-86 PMC1208908

[4] Startzell JK, Owens DA, Mulfinger LM, Cavanagh PR. Stair negotiation in older people: a review. Journal of the American Geriatrics Society. 2000;48(5):567–80. . 10811553 10.1111/j.1532-5415.2000.tb05006.x

[5] Sheehan RC, Gottschall JS. Stair walking transitions are an anticipation of the next stride. Journal of electromyography and kinesiology: official journal of the International Society of Electrophysiological Kinesiology. 2011;21(3):533–41. doi: 10.1016/j.jelekin.2011.01.007 . 21377380 10.1016/j.jelekin.2011.01.007

[6] Startzell JK, Owens DA, Mulfinger LM, Cavanagh PR. Stair negotiation in older people: a review. Journal of the American Geriatrics Society. 2000;48(5):567–80. 10811553 10.1111/j.1532-5415.2000.tb05006.x

[7] Brechter JH, Powers CM. Patellofemoral joint stress during stair ascent and descent in persons with and without patellofemoral pain. Gait Posture. 2002;16(2):115–23. doi: Pii S0966-6362(02)00090-5 doi: 10.1016/S0966-6362(02)00090-5. PubMed PMID: WOS:000178902200002. 12297253 10.1016/s0966-6362(02)00090-5

[8] Herdman SJ, Blatt P, Schubert MC, Tusa RJ. Falls in patients with vestibular deficits. The American journal of otology. 2000;21(6):847–51. Epub 2000/11/15. . 11078074

[9] Agrawal Y, Migliaccio AA, Minor LB, Carey JP. Vestibular hypofunction in the initial postoperative period after surgical treatment of superior semicircular canal dehiscence. Otology & neurotology: official publication of the American Otological Society, American Neurotology Society [and] European Academy of Otology and Neurotology. 2009;30(4):502–6. doi: 10.1097/MAO.0b013e3181a32d69 . 10.1097/MAO.0b013e3181a32d69 19339908

[10] Herdman SJ, Blatt P, Schubert MC, Tusa RJ. Falls in patients with vestibular deficits. American Journal of Otolaryngology 2000;21(6):847–51. Epub 2000/11/15. . 11078074

[11] Cohen H. Vestibular rehabilitation reduces functional disability. Otolaryngology—head and neck surgery: official journal of American Academy of Otolaryngology-Head and Neck Surgery. 1992;107(5):638–43. Epub 1992/11/01. doi: 10.1177/019459989210700505 . 1437201 10.1177/019459989210700505

[12] Svanstrom L. Falls on stairs: an epidemiological accident study. Scandinavian journal of social medicine. 1974;2(3):113–20. . 4432054 10.1177/140349487400200302

[13] Swanenburg J, Zurbrugg A, Straumann D, Hegemann SCA, Palla A, de Bruin ED. A pilot study investigating the association between chronic bilateral vestibulopathy and components of a clinical functional assessment tool. Physiother Theory Pract. 2017;33(6):454–61. doi: 10.1080/09593985.2017.1323362 . 28594306 10.1080/09593985.2017.1323362

[14] Gazzola JM, Gananca FF, Aratani MC, Perracini MR, Gananca MM. Circumstances and consequences of falls in elderly people with vestibular disorder. Braz J Otorhinolaryngol. 2006;72(3):388–92. . 17119777 10.1016/S1808-8694(15)30974-5 PMC9500538

[15] Horak FB. Postural compensation for vestibular loss. Ann N Y Acad Sci. 2009;1164:76–81. doi: 10.1111/j.1749-6632.2008.03708.x ; PubMed Central PMCID: PMC3224857. 19645883 10.1111/j.1749-6632.2008.03708.x PMC3224857

[16] Lacour M, Bernard-Demanze L. Interaction between Vestibular Compensation Mechanisms and Vestibular Rehabilitation Therapy: 10 Recommendations for Optimal Functional Recovery. Front Neurol 2014; 5: 285 2014;5. 25610424 10.3389/fneur.2014.00285 PMC4285093

[17] Timmis MA, Bennett SJ, Buckley JG. Visuomotor control of step descent: evidence of specialised role of the lower visual field. Experimental brain research. 2009;195(2):219–27. doi: 10.1007/s00221-009-1773-x . 19333588 10.1007/s00221-009-1773-x

[18] Zietz D, Hollands M. Gaze behavior of young and older adults during stair walking. Journal of motor behavior. 2009;41(4):357–65. doi: 10.3200/JMBR.41.4.357-366 . 19508962 10.3200/JMBR.41.4.357-366

[19] Miyasike-daSilva V, Allard F, McIlroy WE. Where do we look when we walk on stairs? Gaze behaviour on stairs, transitions, and handrails. Experimental brain research. 2011;209(1):73–83. doi: 10.1007/s00221-010-2520-z . 21188360 10.1007/s00221-010-2520-z

[20] Peng J, Fey NP, Kuiken TA, Hargrove LJ. Anticipatory kinematics and muscle activity preceding transitions from level-ground walking to stair ascent and descent. Journal of biomechanics. 2016;49(4):528–36. Epub 2016/02/03. doi: 10.1016/j.jbiomech.2015.12.041 . 26830440 10.1016/j.jbiomech.2015.12.041

[21] MacDougall HG, Weber KP, McGarvie LA, Halmagyi GM, Curthoys IS. The video head impulse test: diagnostic accuracy in peripheral vestibulopathy. Neurology. 2009;73(14):1134–41. Epub 2009/10/07. doi: 10.1212/WNL.0b013e3181bacf85 ; PubMed Central PMCID: PMC2890997. 19805730 10.1212/WNL.0b013e3181bacf85 PMC2890997

[22] Lee HJ, Chou LS. Balance control during stair negotiation in older adults. Journal of biomechanics. 2007;40(11):2530–6. doi: 10.1016/j.jbiomech.2006.11.001 . 17239890 10.1016/j.jbiomech.2006.11.001

[23] Lay AN, Hass CJ, Gregor RJ. The effects of sloped surfaces on locomotion: a kinematic and kinetic analysis. Journal of biomechanics. 2006;39(9):1621–8. doi: 10.1016/j.jbiomech.2005.05.005 . 15990102 10.1016/j.jbiomech.2005.05.005

[24] Kawamura K, Tokuhiro A, Takechi H. Gait analysis of slope walking: a study on step length, stride width, time factors and deviation in the center of pressure. Acta medica Okayama. 1991;45(3):179–84. doi: 10.18926/AMO/32212 . 1891977 10.18926/AMO/32212

[25] Jacobs JV. A review of stairway falls and stair negotiation: Lessons learned and future needs to reduce injury. Gait Posture. 2016;49:159–67. doi: 10.1016/j.gaitpost.2016.06.030 . 27427833 10.1016/j.gaitpost.2016.06.030

[26] Kasneci E, Black AA, Wood JM. Eye-Tracking as a Tool to Evaluate Functional Ability in Everyday Tasks in Glaucoma. J Ophthalmol. 2017;2017:6425913 doi: 10.1155/2017/6425913 ; PubMed Central PMCID: PMCPMC5331274 publication of this paper. 28293433 10.1155/2017/6425913 PMC5331274

[27] Tapiro H, Borowsky A, Oron-Gilad T, Parmet Y. Where do older pedestrians glance before deciding to cross a simulated two-lane road? A pedestrian simulator paradigm. Proceedings of the Human Factors and Ergonomics Society Annual Meeting. 2016;60(1):11–5. doi: https://doi.org/10.1177/1541931213601003

[28] Sotirakis H, Kyvelidou A, Mademli L, Stergiou N, Hatzitaki V. Aging affects postural tracking of complex visual motion cues. Experimental brain research. 2016;234(9):2529–40. doi: 10.1007/s00221-016-4657-x ; PubMed Central PMCID: PMCPMC5253232. 27126061 10.1007/s00221-016-4657-x PMC5253232

[29] Adelsberger R, Valko Y, Straumann D, Troster G. Automated Romberg testing in patients with benign paroxysmal positional vertigo and healthy subjects. IEEE transactions on bio-medical engineering. 2015;62(1):373–81. doi: 10.1109/TBME.2014.2354053 . 25203981 10.1109/TBME.2014.2354053

[30] Adelsberger A, Tröster G. Unobtrusive assessment of bipedal balance performance. 8th International Conference on Body Area Networks; September 30—October 02; Boston, Massachusetts: EDUL; 2013. p. 229–32.

[31] Field A. Discovering Statistics Using SPSS. Third ed. Los Angeles: Sage; 2009.

[32] Horak FB. Postural compensation for vestibular loss and implications for rehabilitation. Restor Neurol Neurosci. 2010;28(1):57–68. doi: 10.3233/RNN-2010-0515 ; PubMed Central PMCID: PMC2965039. 20086283 10.3233/RNN-2010-0515 PMC2965039

[33] Miyasike-daSilva V, McIlroy WE. Does it really matter where you look when walking on stairs? Insights from a dual-task study. PLoS One. 2012;7(9):e44722 doi: 10.1371/journal.pone.0044722 ; PubMed Central PMCID: PMCPMC3435292. 22970297 10.1371/journal.pone.0044722 PMC3435292

[34] Den Otter AR, Hoogwerf M, Van Der Woude LH. The role of tread fixations in the visual control of stair walking. Gait Posture. 2011;34(2):169–73. doi: 10.1016/j.gaitpost.2011.04.004 . 21550247 10.1016/j.gaitpost.2011.04.004

[35] Corbetta M, Akbudak E, Conturo TE, Snyder AZ, Ollinger JM, Drury HA, et al A common network of functional areas for attention and eye movements. Neuron. 1998;21(4):761–73. . 9808463 10.1016/s0896-6273(00)80593-0

[36] Adolphe RM, Vickers JN, Laplante G. The effects of training visual attention on gaze behaviour and accurcy: A pilot Study. International Journal of Sports Vision. 1997;4(1):28–33.

[37] Young WR, Hollands MA. Can telling older adults where to look reduce falls? Evidence for a causal link between inappropriate visual sampling and suboptimal stepping performance. Experimental brain research. 2010;204(1):103–13. doi: 10.1007/s00221-010-2300-9 . 20512484 10.1007/s00221-010-2300-9

[38] Swanenburg J, Hegemann SC, Zurbrugg A, Palla A, de Bruin ED. Reliability and validity of the extended timed-get-up-and-go test in patients with bilateral vestibular loss. NeuroRehabilitation. 2014;34(4):799–807. doi: 10.3233/NRE-141083 . 24796440 10.3233/NRE-141083

[39] Schmidheiny A, Swanenburg J, Straumann D, de Bruin ED, Knols RH. Discriminant validity and test re-test reproducibility of a gait assessment in patients with vestibular dysfunction. BMC Ear Nose Throat Disord. 2015;15:6 doi: 10.1186/s12901-015-0019-8 ; PubMed Central PMCID: PMCPMC4619276. 26500447 10.1186/s12901-015-0019-8 PMC4619276

[40] Gill-Body KM, Krebs DE, Parker SW, Riley PO. Physical therapy management of peripheral vestibular dysfunction: two clinical case reports. Physical Therapy. 1994;74(2):129–42. Epub 1994/02/01. . 8290618 10.1093/ptj/74.2.129

[41] Krebs DE, Gill-Body KM, Parker SW, Ramirez JV, Wernick-Robinson M. Vestibular rehabilitation: useful but not universally so. Otolaryngology-Head and Neck Surgery. 2003;128(2):240–50. Epub 2003/02/26. doi: 10.1067/mhn.2003.72 S0194599802232143 [pii]. . 12601321 10.1067/mhn.2003.72

[42] Brandt T, Strupp M, Benson J. You are better off running than walking with acute vestibulopathy. Lancet. 1999;354(9180):746 Epub 1999/09/04. doi: 10.1016/S0140-6736(99)03179-7 . 10475195 10.1016/S0140-6736(99)03179-7

[43] Schniepp R, Wuehr M, Neuhaeusser M, Kamenova M, Dimitriadis K, Klopstock T, et al Locomotion speed determines gait variability in cerebellar ataxia and vestibular failure. Movement Disorders. 2012;27(1):125–31. Epub 2011/10/15. doi: 10.1002/mds.23978 . 21997342 10.1002/mds.23978

[44] Jahn K, Strupp M, Brandt T. Both actual and imagined locomotion suppress spontaneous vestibular nystagmus. Neuroreport. 2002;13(16):2125–8. Epub 2002/11/20. . 12438939 10.1097/00001756-200211150-00027

[45] Mori S, Matsui T, Kuze B, Asanome M, Nakajima K, Matsuyama K. Cerebellar-induced locomotion: reticulospinal control of spinal rhythm generating mechanism in cats. Annals of the New York Academy of Sciences. 1998;860:94–105. Epub 1999/02/03. . 9928304 10.1111/j.1749-6632.1998.tb09041.x

[46] Prentice SD, Hasler EN, Groves JJ, Frank JS. Locomotor adaptations for changes in the slope of the walking surface. Gait Posture. 2004;20(3):255–65. doi: 10.1016/j.gaitpost.2003.09.006 . 15531172 10.1016/j.gaitpost.2003.09.006

[47] Stuart S, Alcock L, Godfrey A, Lord S, Rochester L, Galna B. Accuracy and re-test reliability of mobile eye-tracking in Parkinson's disease and older adults. Med Eng Phys. 2016;38(3):308–15. doi: 10.1016/j.medengphy.2015.12.001 . 26786676 10.1016/j.medengphy.2015.12.001

[48] Aquaroni Ricci N, Aratani MC, Caovilla HH, Freitas Gananca F. Effects of conventional versus multimodal vestibular rehabilitation on functional capacity and balance control in older people with chronic dizziness from vestibular disorders: design of a randomized clinical trial. Trials. 2012;13:246 doi: 10.1186/1745-6215-13-246 ; PubMed Central PMCID: PMC3551791. 23276084 10.1186/1745-6215-13-246 PMC3551791

